# Holographic Dark Energy in Modified Barrow Cosmology

**DOI:** 10.3390/e25040569

**Published:** 2023-03-26

**Authors:** Ahmad Sheykhi, Maral Sahebi Hamedan

**Affiliations:** 1 Department of Physics, College of Sciences, Shiraz University, Shiraz 71454, Iran; 2 Biruni Observatory, College of Sciences, Shiraz University, Shiraz 71454, Iran

**Keywords:** dark energy, thermodynamics-gravity conjecture, barrow entropy, cosmology

## Abstract

Thermodynamics–gravity conjecture implies that there is a deep connection between the gravitational field equations and the first law of thermodynamics. Therefore, any modification to the entropy expression directly modifies the field equations. By considering the modified Barrow entropy associated with the apparent horizon, the Friedmann equations are modified as well. In this paper, we reconsider the holographic dark energy (HDE) model when the entropy is in the form of Barrow entropy. This modification to the entropy not only changes the energy density of the HDE but also modifies the Friedmann equations. Therefore, one should take into account the modified HDE in the context of modified Friedmann equations. We study the Hubble horizon and the future event horizon as IR cutoffs and investigate the cosmological consequences of this model. We also extend our study to the case where dark matter (DM) and dark energy (DE) interact with each other. We observe that Barrow exponent δ significantly affects the cosmological behavior of HDE, and in particular, the equation of state (EoS) parameter can cross the phantom line $\left(w_{de}<-1\right)$. Additionally, adding δ remarkably affects the deceleration parameter and shifts the time of universe phase transition.

## 1. Introduction

A huge number of cosmological observations support that our universe is currently experiencing a phase of accelerated expansion [1,2,3]. Understanding the cause of acceleration of the cosmic expansion has been one of the primarily unsolved problems in modern cosmology. The component which is responsible for this acceleration is usually dubbed DE, and disclosing its nature and origin has been on the hottest topics of research in the past two decades (see [4] for a comprehensive review). The simplest possible candidate for DE is the cosmological constant, denoted as Λ in Einstein’s field equations of general relativity. The resulting model is termed ΛCDM, which is the most acceptable model for describing accelerated expansion as well as solving the DM puzzle. Nevertheless, recent observations have indicated tension with the ΛCDM model and revealed that ΛCDM is not the best fit for some data sets [5]. Many alternative theories have been proposed to modify the matter and energy sector of Einstein’s field equations. Among these proposals, there is a supposition which plays an important role in finding the nature of DE based on the holographic principle [6,7,8], and it is known as holographic DE (HDE) [9]. This model is based on the fact that the entropy associated with the boundary is proportional to the area which was first pointed out by Bekenstein and Hawking for black holes [10]. According to the Bekenstein–Hawking area law, the entropy of a black hole is given by

(1) $$S=\frac{A}{4L_{p}^{2}},$$

where *A* is the horizon area and $L_{p}^{2}$ denotes the Planck area. HDE models have received a lot of attention in the literature [11,12,13,14,15,16,17,18,19,20,21,22,23,24,25,26,27,28].

Inspired by the COVID-19 virus structure, J. D. Barrow argued that quantum gravitational effects may deform the geometry of the black hole horizon, leading to intricate fractal features [29]. He discussed how the area law of the black hole entropy is modified, given by

(2) $$\begin{array}{c} S={\left(\frac{A}{A_{0}}\right)}^{1+\delta /2}, \end{array}$$

where *A* is the black hole horizon area and $A_{0}$ is the Planck area. The exponent δ’s range is 0≤δ≤1 and represents the amount of quantum gravitational deformation effects. The area law is reproduced in the case of δ=0, and $A_{0}\to 4L_{p}^{2}=4G$. On the other hand, the most intricate and fractal structure of the horizon is obtained by δ=1. In the cosmological set-up, the effects of Barrow entropy on the cosmic evolution have been investigated from different viewpoints. For example, modification of the area law leads to a new HDE model based on Barrow entropy [30,31]. On the other hand, it was recently proven that Barrow entropy as well as any other known entropy (Tsalis, Renyi, Kaniadakis, etc.) is just a sub-case of the generalized entropy expression introduced in [32,33]. Other studies on the cosmological consequences of the Barrow entropy were carried out in [34,35,36,37,38,39,40,41,42,43,44].

Nowadays, it is a general belief that there is a deep correspondence between the gravitational field equations and the laws of thermodynamics (see [45,46,47,48,49,50,51,52,53,54,55,56,57,58] and the references therein). In the background of cosmology, “thermodynamics-gravity” conjecture translates to the correspondence between Friedmann equations describing the evolution of the universe and the first law of thermodynamics on the apparent horizon. It has been confirmed that one can always rewrite the Friedmann equations in any gravity theory in the form of the first law of themodynamics on the apparent horizon, and vice versa [59,60,61,62].

In the present work, we are going to investigate HDE when the entropy associated with the apparent horizon is in the form of Barrow enetropy, given in Equation (2). According to the thermodynamics-gravity conjecture, any modification to the entropy expression leads to modified Friedmann equations. The modified Friedmann equations through Barrow entropy were introduced in [63,64]. A cosmological scenario based on Barrow entropy was introduced in [65], where it was argued that the exponent δ cannot reproduce any term which may play the role of DE, and one still needs to take into account the DE component in the modified Friedmann equations to reproduce the accelerated universe. On the other hand, the HDE density, which is based on the holographic principle, is modified due to the modification of the entropy. Although the authors of [30] modified the energy density of HDE, they still performed their calculations in the set-up of standard Friedmann equations. This is indeed inconsistent with the thermodynamics-gravity conjecture, which states that any modification to the entropy should modify the field equations of gravity. In other words, one should consider a modified HDE density in the background of modified Friedmann equations. Our work differs from [30,31,38] in that we modify both the energy density of HDE as well as the Friedmann equations describing the evolution of the universe. Throughout this paper, we set $\kappa_{B}=1=c=\hbar$ for simplicity.

This paper is outlined as follows. In Section 2, we study HDE in Barrow cosmology when the IR cutoff is the Hubble radius. In Section 3, we consider the future event horizon as the IR cutoff and explore the cosmological consequences of HDE through modified cosmology based on Barrow entropy. The last section is devoted to the closing remarks.

## 2. HDE with the Hubble Horizon as the IR Cutoff

We consider a homogeneous and isotropic flat universe which is described by the line element

(3) $$ds^{2}=-dt^{2}+a^{2}\left(t\right)\delta_{ij}dx^{i}dx^{j},$$

where a(t) is the scale factor. By applying Barrow entropy (Equation (2)) to the holographic framework, one can obtain the HDE density in the form of [30]

(4) $$\rho_{de}=CL^{\delta -2},$$

where *L* is the holographic horizon length and *C* is a parameter with the dimension ${\left[L\right]}^{-2-\delta }$. For the latter’s convenience, we chose $C=3c^{2}M_{\mathrm{eff}}^{2}$, where $c^{2}$ is the holographic model parameter of the first order [12] and $M_{\mathrm{eff}}^{2}$ is the effective Planck mass, which we shall introduce latter. Note that in the case where δ=0, we have $M_{\mathrm{eff}}^{2}\to M_{p}^{2}$, and the standard HDE density $\rho_{de}=3c^{2}M^{2}/L^{2}$ is restored.

The modified Friedmann equation based on Barrow entropy in a flat universe is given by [64,65]

(5) $$H^{2-\delta }=\frac{8\pi G_{\mathrm{eff}}}{3}\left(\rho_{m}+\rho_{de}\right),$$

where $H=\dot{a}/a$ is the Hubble parameter and $\rho_{m}$ and $\rho_{de}$ are the energy density of pressureless matter and DE, respectively. Here, $G_{\mathrm{eff}}$ stands for the effective Newtonian gravitational constant [64]

(6) $$G_{\mathrm{eff}}\equiv \frac{A_{0}}{4}\left(\frac{2-\delta }{2+\delta }\right){\left(\frac{A_{0}}{4\pi }\right)}^{\delta /2}.$$

If we define $M_{\mathrm{eff}}^{2}={\left(8\pi G_{\mathrm{eff}}\right)}^{-1}$, then the Friedmann Equation (5) can be written as

(7) $$H^{2-\delta }=\frac{1}{3M_{\mathrm{eff}}^{2}}\left(\rho_{m}+\rho_{de}\right),$$

By taking the Hubble radius as the IR cutoff $L=H^{-1}$, the HDE density can be written as

(8) $$\rho_{de}=3c^{2}M_{\mathrm{eff}}^{2}H^{2-\delta },$$

In the remaining part of this section, we shall consider the case without interaction between dark sectors and the interacting case separately.

### 2.1. Non-Interacting Case

When two dark components of the universe evolve separately, they satisfy two independent energy conservation equations:

(9) $$\begin{array}{ccc} & & {\dot{\rho }}_{de}+3H\left(1+w_{de}\right)\rho_{de}=0, \end{array}$$

(10) $$\begin{array}{ccc} & & {\dot{\rho }}_{m}+3H\rho_{m}=0, \end{array}$$

where $w_{de}=p_{de}/\rho_{de}$ stands for the EoS parameter of HDE. It is also convenient to introduce the dimensionless density parameters as follows:

(11) $$\begin{array}{ccc} & & \Omega_{de}=\frac{\rho_{de}}{\rho_{c}}=\frac{\rho_{de}}{3M_{\mathrm{eff}}^{2}H^{2-\delta }}, \end{array}$$

(12) $$\begin{array}{ccc} & & \Omega_{m}=\frac{\rho_{m}}{\rho_{c}}=\frac{\rho_{m}}{3M_{\mathrm{eff}}^{2}H^{2-\delta }}, \end{array}$$

Thus, the Friedmann equation (Equation (7)) can be written as

(13) $$\begin{array}{ccc} & & \Omega_{m}+\Omega_{de}=1, \end{array}$$

where $\rho_{c}=3M_{\mathrm{eff}}^{2}H^{2-\delta }$ is the effective critical energy density. By substituting Equation (8) into Equation (11), one finds that $\Omega_{de}=c^{2}$. By taking the time derivative of the Friedmann equation (Equation (7)) and using the continuity equations (Equations (9) and (10)), we arrive at

(14) $$\frac{\dot{H}}{H^{2}}=-\frac{3}{2-\delta }\left(1+\Omega_{de}w_{de}\right).$$

If we take the derivative of Equation (8) with respect to cosmic time and use Equations (9) and (14), we find that $w_{de}=0$. This result is similar to HDE with the Hubble horizon as the IR cutoff in standard cosmology and cannot lead to an accelerated universe [12,15].

### 2.2. Interacting Case

When the two dark sectors of the universe interact with each other, they do not satisfy the independent conservation equations. Instead, they satisfy the semi-energy conservation equations as follows:

(15) $$\begin{array}{ccc} & & {\dot{\rho }}_{de}+3H\left(1+w_{de}\right)\rho_{de}=-Q, \end{array}$$

(16) $$\begin{array}{ccc} & & {\dot{\rho }}_{m}+3H\rho_{m}=Q, \end{array}$$

where $Q=3b^{2}H\rho_{de}$ represents the interaction term. By taking the time derivative of Equation (8) and using Equations (14) and (15), we find

(17) $$w_{de}=-\frac{b^{2}}{1-c^{2}},$$

which is independent of the exponent δ and is similar to the case of standard cosmology [15,23]. In order to reach $w_{de}<0$, we should have $c^{2}<1$. That aside, for $b^{2}=1-c^{2}$, this model mimics the cosmological constant. Moreover, for $b^{2}>1-c^{2}$, this model can cross the phantom line. The EoS parameter of total energy and matter is defined by

(18) $$w_{\mathrm{tot}}=\Omega_{de}w_{de}=-\frac{b^{2}c^{2}}{1-c^{2}}.$$

It was argued that in modified Barrow cosmology, the condition for the accelerated expansion reads as follows [64]:

(19) $$w_{\mathrm{tot}}<-\frac{\left(1+\delta \right)}{3}.$$

Combining the condition in Equation (19) with Equation (18) implies $3b^{2}c^{2}>\left(1+\delta \right)\left(1-c^{2}\right)$ for an accelerated universe. The deceleration parameter can be easily derived:

(20) $$\begin{array}{ccc} q & = & -1-\frac{\dot{H}}{H^{2}}=-1+\frac{3}{2-\delta }\left[1+\Omega_{de}w_{de}\right]\\ & & =-1+\frac{3}{2-\delta }\left(1-\frac{b^{2}c^{2}}{1-c^{2}}\right). \end{array}$$

which is a constant but depends on δ. In an accelerating universe, q<0, which translates to $3b^{2}c^{2}>\left(1+\delta \right)\left(1-c^{2}\right)$ and is consistent with the previous condition in Equation (19).

## 3. HDE with the Future Event Horizon as the IR Cutoff

In this section, we consider the future event horizon as the IR cutoff: [12]

(21) $$L=R_{h}=a\int_{t}^{\infty }\frac{dt}{a}=a\int_{t}^{\infty }\frac{da}{Ha^{2}},$$

Therefore, the HDE density parameter becomes

(22) $$\Omega_{de}=c^{2}{\left(HR_{h}\right)}^{\delta -2},\Rightarrow HR_{h}={\left(\frac{\Omega_{de}}{c^{2}}\right)}^{1/\left(\delta -2\right)}.$$

By taking the time derivative of Equation (21), we find

(23) $${\dot{R}}_{h}=HR_{h}-1,$$

In addition, taking the time derivative of the energy density $\rho_{de}=3c^{2}M_{\mathrm{eff}}^{2}R_{h}^{\delta -2}$ yields

(24) $${\dot{\rho }}_{de}=3\left(\delta -2\right)c^{2}M_{\mathrm{eff}}^{2}{\dot{R}}_{h}R_{h}^{\delta -3}=\frac{\delta -2}{R_{h}}{\dot{R}}_{h}\rho_{de}.$$

Again, we shall consider the non-interacting and interacting cases separately.

### 3.1. Non-Interacting Case

By combining Equations (9), (23) and (24), one can obtain

(25) $$w_{de}=-\frac{1+\delta }{3}+\frac{\delta -2}{3}{\left(\frac{\Omega_{de}}{c^{2}}\right)}^{\frac{1}{2-\delta }},$$

for the EoS parameter of HDE. Note that for δ=0, one restores the EoS parameter of HDE in standard cosmology [16]. We can also obtain the evolution of the density parameter of HDE. By differentiating $\Omega_{de}$ with respect to time and using the relations in ${\dot{\Omega }}_{de}=H\Omega_{de}^{\prime }$ and Equation (14), we arrive at

(26) $$\Omega_{de}^{\prime }=\Omega_{de}\left\{\left(\delta -2\right)\left[1-{\left(\frac{\Omega_{de}}{c^{2}}\right)}^{\frac{1}{2-\delta }}\right]+3\left(1+\Omega_{de}w_{de}\right)\right\},$$

where the prime denotes the derivative with respect to x=lna. Substituting $w_{de}$ from Equation (25) into the above relation yields

(27) $$\Omega_{de}^{\prime }=\Omega_{de}\left(1-\Omega_{de}\right)\left\{\left(1+\delta \right)+\left(2-\delta \right){\left(\frac{\Omega_{de}}{c^{2}}\right)}^{\frac{1}{2\delta }}\right\}.$$

**Figure 1:**
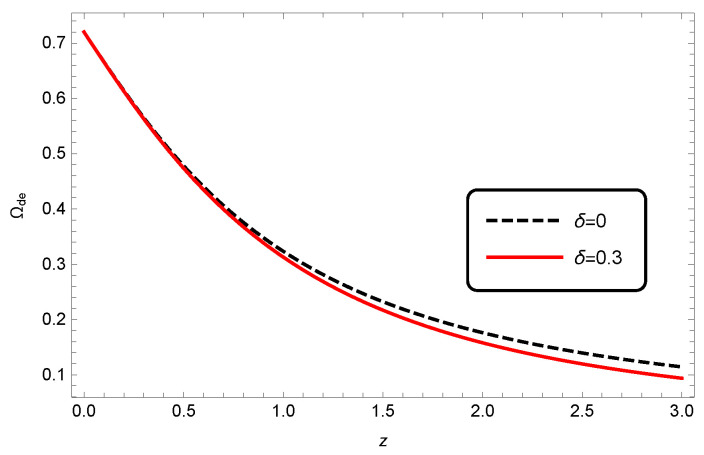
Evolution of $\Omega_{de}$ as a function of redshift *z* for non-interacting HDE. Here, we have set $c^{2}=1$ and $\Omega_{de,0}=0.72$.

This equation shows the evolution of the density parameter of HDE in the context of modified Barrow cosmology. We plotted the evolution of $\Omega_{de}$ in Barrow cosmology in Figure 1. From this figure, we see that at each *z*, the value of $\Omega_{de}$ decreases with increasing δ. Aside from that, the difference between $\Omega_{de}$ in standard cosmology and modified Barrow cosmology increases at higher redshifts.

**Figure 2:**
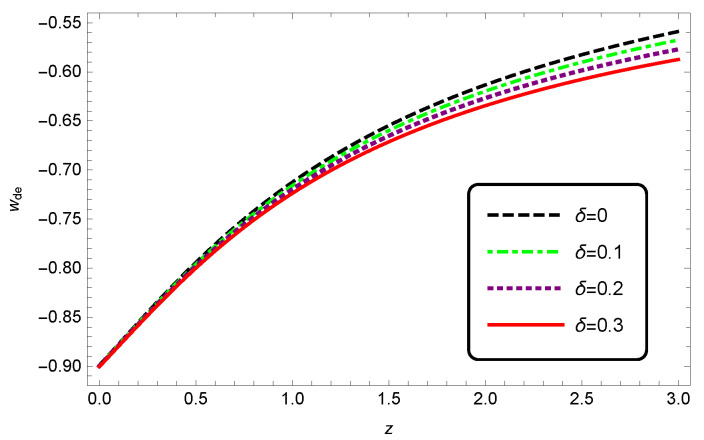
Evolution of $w_{de}$ as a function of redshift *z* for non-interacting HDE. Here, we set $c^{2}=1$, $M_{\mathrm{eff}}^{2}=1$.

The evolution of the EoS parameter $w_{de}$ for HDE versus *z* is plotted in Figure 2 for different values of δ. We see that at the present time, the values of $w_{de}$ is independent of δ, while its behavior differs for the early times. For z=0, we have $w_{de}\approx -0.9$, and increasing δ will decrease $w_{de}$ at all times. For example, for δ=0.3 at z=0, we have $w_{de}=-0.91$, which is closer than the standard model to observational data. Therefore, in modified Barrow cosmology, $w_{de}$ of HDE at the present time is compatible with the observations.

Next, we examine the deceleration parameter $q=-\ddot{a}/\left(aH^{2}\right)$. Using Equations (14) and (25), one finds

(28) $$\begin{array}{ccc} q & = & -1-\frac{\dot{H}}{H^{2}}=-1+\frac{3}{2-\delta }\left(1+\Omega_{de}w_{de}\right)\\ & = & \frac{1+\delta }{2-\delta }\left(1-\Omega_{de}\right)-\Omega_{de}{\left(\frac{\Omega_{de}}{c^{2}}\right)}^{\frac{1}{2-\delta }}. \end{array}$$

**Figure 3:**
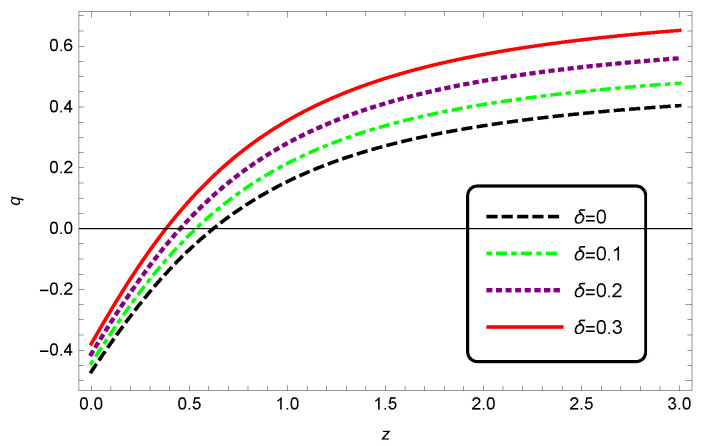
The behavior of the deceleration parameter *q* as a function of redshift *z* for non-interacting HDE in Barrow cosmology, where we set $c^{2}=1$.

The evolutionary behavior of the deceleration parameter is plotted in Figure 3. From this figure, we see that when increasing the Barrow exponent δ, the transition from deceleration to acceleration took place in the lower redshift.

### 3.2. Interacting Case

Next, we investigate the case for an FRW universe filled with HDE and DM exchanging energy. We consider the future event horizon as the IR cutoff. In the interacting case, the conservation equations are in the form of Equations (15) and (16). Following the method of the previous section, we can find the EoS parameter as follows:

(29) $$w_{de}=-b^{2}-\frac{\delta +1}{3}+\frac{\delta -2}{3}{\left(\frac{c^{2}}{\Omega_{de}}\right)}^{\frac{1}{\delta -2}}.$$

In Table 1, we present the numerical results for EoS parameter at the present time (z=0), in case of interacting HDE, in the context of modified Barrow cosmology. In this case, the evolutionary equation for $\Omega_{de}$ becomes

(30) $$\begin{array}{ccc} \Omega_{de}^{\prime } & = & \Omega_{de}\left(1-\Omega_{de}\right)\left\{\left(1+\delta \right)+\left(2-\delta \right){\left(\frac{\Omega_{de}}{c^{2}}\right)}^{\frac{1}{2\delta }}\right\}\\ & & -3b^{2}\Omega_{de}^{2}, \end{array}$$

while the deceleration parameter reads

(31) $$\begin{array}{ccc} & & q=-1-\frac{\dot{H}}{H^{2}}=-1+\frac{3}{2-\delta }\left(1+\Omega_{de}w_{de}\right)\\ & = & \frac{1+\delta }{2-\delta }\left(1-\Omega_{de}\right)-\Omega_{de}{\left(\frac{\Omega_{de}}{c^{2}}\right)}^{\frac{1}{2-\delta }}-3\frac{b^{2}\Omega_{de}}{2-\delta }. \end{array}$$

**Figure 4:**
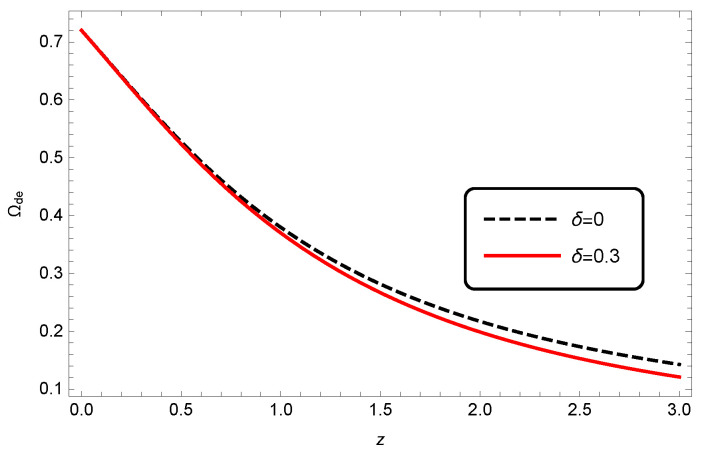
Evolution of $\Omega_{de}$ as a function of redshift *z* for interacting HDE in Barrow cosmology. Here, we take $c^{2}=1$, $M_{\mathrm{eff}}^{2}=1$ and $b^{2}=0.1$.

We plotted the evolution of $\Omega_{de}$ for different values of δ in Figure 4.

**Figure 5:**
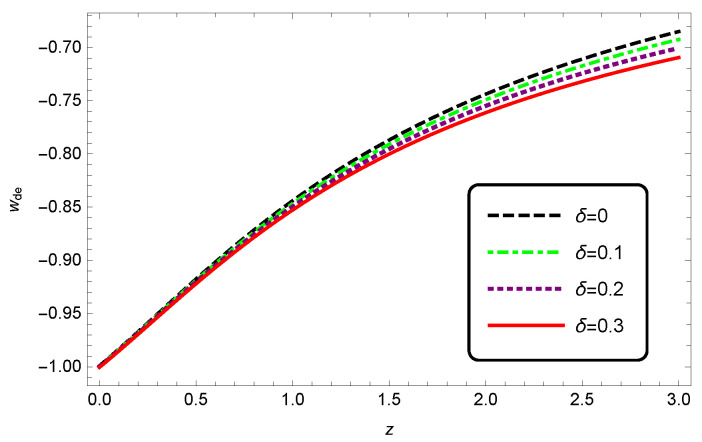
The evolution of EoS parameter $w_{de}$ as a function of redshift *z* for interacting HDE. Here, we take $c^{2}=1$, $M_{\mathrm{eff}}^{2}=1$ and $b^{2}=0.1$.

**Figure 6:**
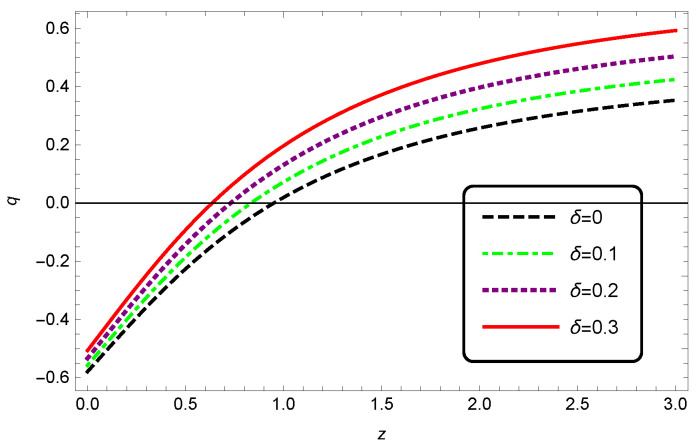
Evolution of the deceleration parameter *q* as a function of redshift *z* for interacting HDE. We have taken $c^{2}=1$, $M_{\mathrm{eff}}^{2}=1$ and $b^{2}=0.1$.

We can also plot the EoS and deceleration parameters for the interacting HDE in modified Barrow cosmology. From Figure 5, we see that with increasing δ values, the EoS parameter decreased as well. We also observed from Figure 6 that when increasing the Barrow exponent δ, the transition from deceleration to acceleration occurred at a lower redshift.

**Figure 7:**
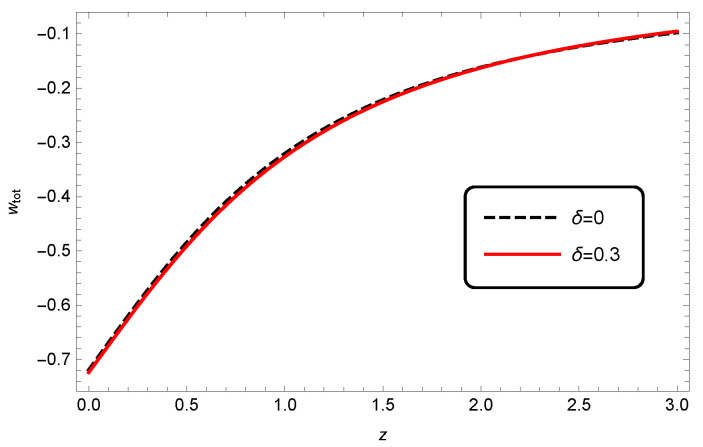
Evolution of $w_{tot}$ as a function of redshift *z* for interacting HDE, where $c^{2}=1$, $M_{\mathrm{eff}}^{2}=1$ and $b^{2}=0.1$.

The evolution of the total EoS parameter for different values of δ in Figure 7 indicates that there was no significant difference between $w_{tot}$ of HDE in standard cosmology (δ=0) and in modified Barrow cosmology (δ≠0).

**Figure 8:**
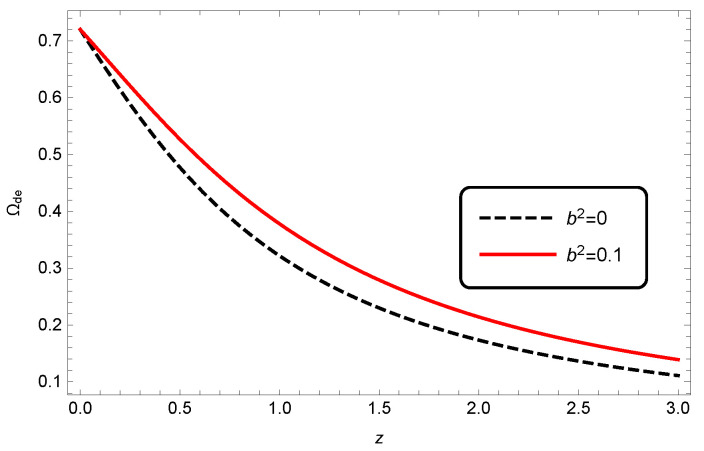
Evolution of $\Omega_{de}$ versus *z* for modified Barrow HDE, where $c^{2}=1$, $M_{\mathrm{eff}}^{2}=1$ and δ=0.2.

By fixing the Barrow component δ and changing the values of $b^{2}$, we can reveal the effects of the interaction term on the cosmological behavior of our model. In Figure 8, we plotted the evolution of $\Omega_{de}$ for interacting HDE in modified Barrow cosmology. It was seen that at each time, the value of $\Omega_{de}$ was higher compared with standard cosmology.

**Figure 9:**
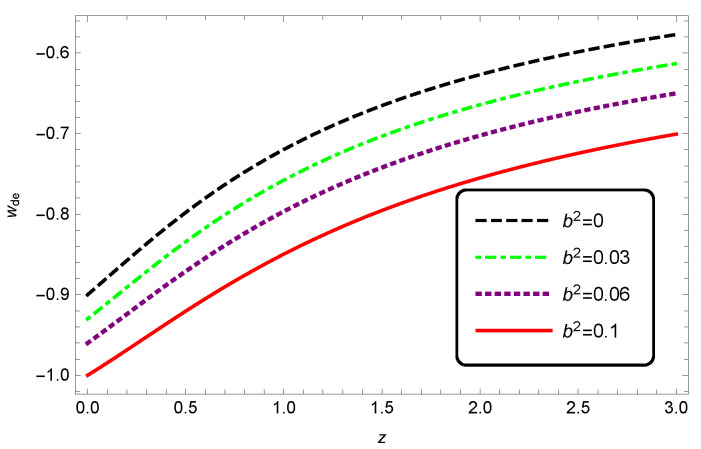
Evolution of EoS parameter $w_{de}$ as a function of redshift *z* for interacting HDE. Here, we take $c^{2}=1$, $M_{\mathrm{eff}}^{2}=1$ and δ=0.2.

In Figure 9, we can see that $w_{de}$ of interacting HDE decreased with an increasing coupling constant $b^{2}$. For δ=0.2 and $b^{2}=0.1$, we see that $w_{de}$ crossed the phantom regime.

**Figure 10:**
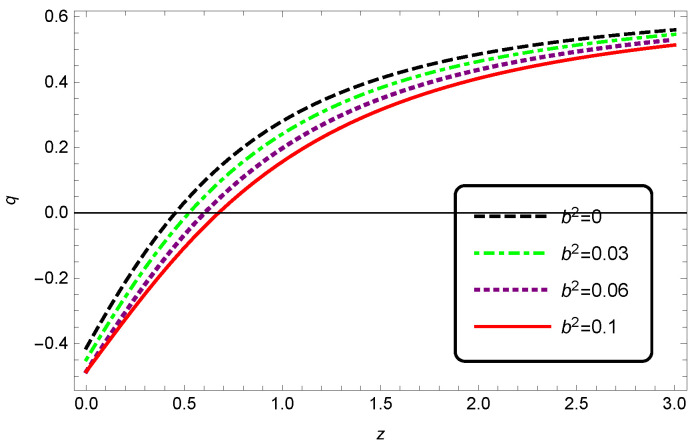
Evolution of the deceleration parameter *q* as a function of redshift *z* for interacting HDE in modified Barrow cosmology. Here, we have taken $c^{2}=1$, $M_{\mathrm{eff}}^{2}=1$ and δ=0.2.

As one can see in Figure 10, changing $b^{2}$ affected the phase transition of interacting HDE in Barrow cosmology. We observe that unlike the effects of adding the values of δ on the deceleration parameter, increasing $b^{2}$ would shift the transition to higher redshifts, and this means that the expansion of the universe changed from a decelerating phase to an accelerating phase at much earlier times. By comparing the effects of changing δ and $b^{2}$, given by Figure 6 and Figure 10, respectively, we can see that the best values for the parameters were in the ranges of $0.06<b^{2}<0.1$ and 0.2<δ<0.3.

**Figure 11:**
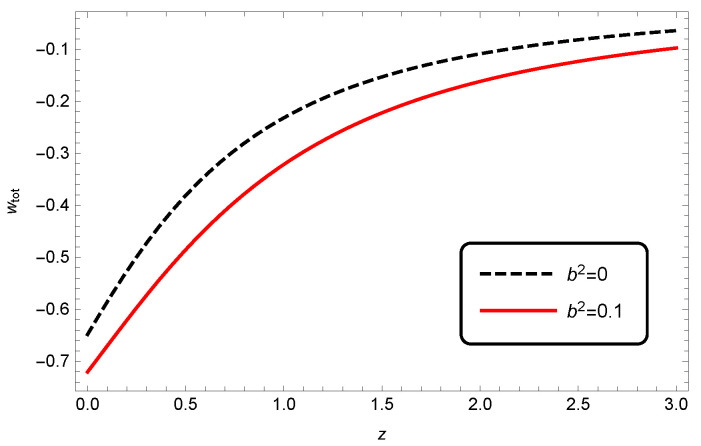
Evolution of $w_{tot}$ as a function of redshift *z* for interacting HDE in modified Barrow cosmology. Here, we have taken $c^{2}=1$, $M_{\mathrm{eff}}^{2}=1$ and δ=0.2.

Again, by using our previous results for $\Omega_{de}$ and $w_{de}$, we could plot the total EoS parameter for different values of $b^{2}$ in the case of interacting HDE, which is shown in Figure 11. Adding a coupling constant would decrease $w_{tot}$ significantly during all times and reveal the difference between interacting and non-interacting HDE in the framework of Barrow cosmology.

## 4. Closing Remarks

According to thermodynamics-gravity conjecture, any modification to the entropy modifies the energy density of HDE as well as the Friedmann equations. Based on this, and using the modified Friedmann equations, we investigated HDE when the entropy was in the form of Barrow entropy. We first took the Hubble radius as the IR cutoff and showed that an accelerated universe could be achieved only in the interacting case. This behavior is similar to HDE in standard cosmology with a Hubble cutoff. We then considered the future event horizon as the IR cutoff and investigated both non-interacting and interacting HDE in a flat universe.

We examined the effects of the Barrow exponent δ on the cosmological evolution of HDE. We observed that for δ=0, the EoS parameter of non-interacting HDE lies completely in the quintessence regime, while for the interacting case, it can cross the phantom line. In both cases, with increasing δ values, the EoS parameter $w_{de}$ decreased at any time. Another interesting result we found in this work is that the presence of δ can change the time of phase transition of the universe from deceleration to acceleration. Indeed, with increasing δ values, the phase transition occurred at lower redshifts. This behavior was seen for both the non-interacting and interacting cases. On the other hand, for a fixed value of δ, if we increased the coupling constant of the interaction term, the transition occurred in a higher redshift.

To sum up, the incorporation of modified Friedmann equations into HDE improved the phenomenology compared with the standard Friedmann equation while keeping the Barrow exponent δ at smaller values. This is an advantage of this scenario, since in a more realistic case, we expect the Barrow exponent to be closer to the standard Bekenstein–Hawking value and have the results compatible with the observations.

## Figures and Tables

**Table 1:** Numerical results for EoS parameter with interacting HDE at the present time z=0.

$w_{\mathrm{de}}$	$b^{2}=0.03$	$b^{2}=0.06$	$b^{2}=0.1$
δ=0	−0.92909	−0.95909	−0.99909
δ=0.1	−0.92944	−0.95944	−0.99944
δ=0.2	−0.92991	−0.95991	−0.99991
δ=0.3	−0.93049	−0.96049	−1.00049

## Data Availability

No data are associated in the manuscript.
